# Safety and immunogenicity of Ad5-nCoV administered intradermally by needle-free injector in rats

**DOI:** 10.3389/fmed.2025.1543398

**Published:** 2025-02-14

**Authors:** Li Chen, Xiaoyin Zhang, Lairun Jin, Lihua Hou, Fengcai Zhu, Jingxin Li

**Affiliations:** ^1^School of Public Health, Southeast University, Nanjing, Jiangsu, China; ^2^Jiangsu Provincial Medical Innovation Center, National Health Commission Key Laboratory of Enteric Pathogenic Microbiology, Jiangsu Provincial Center for Disease Control and Prevention, Nanjing, China; ^3^Taizhou Center for Disease Control and Prevention, Taizhou, Jiangsu, China; ^4^Beijing Institute of Biotechnology, Academy of Military Medical Sciences, Beijing, China

**Keywords:** COVID-19, adenovirus type-5 vector-based COVID-19 vaccine, immunogenicity, safety, needle-free injector

## Abstract

**Objectives:**

To evaluate the safety and immunogenicity of adenovirus type 5 vectored COVID-19 vaccine (Ad5-nCoV), by intradermal immunization with a needle-free injector in rats.

**Methods:**

This study was divided into two parts. In study A, 105 rats were randomly assigned to seven groups, to receive the low-dose, medium-dose, or high-dose vaccine by needle-free intradermal injections (NFI), or needle-based intramuscular injections (NI), or needle-free intradermal injections with saline solution as a control group. Blood samples were collected on day 0 before vaccination, and day 7, day 14, day 21 and day 28 after vaccination. Binding antibody, pseudovirus neutralizing antibody as well as cellular immune response were measured. The safety endpoints included weight changes and skin reactions. In study B, 32 rats were randomly assigned to four groups to receive low-dose, or medium-dose vaccine by NFI or NI, to observe pathological changes at the injection site following immunization.

**Results:**

No safety concern was noted associated with NFI of Ad5-nCoV. Comparable levels of neutralizing antibodies against various variants induced by NFI compared to NI at the same dosage.

**Conclusion:**

The NFI immunization would be considered as an alternative immunization method to replace the traditional NI for the Ad5-nCoV.

## 1 Introduction

The administration of COVID-19 vaccine was predominated by the intramuscular injection. However, empirical evidence has demonstrated that intradermal injection routes are more immunogenic with lower dosage compared to intramuscular injection (1, 2). The skin is regarded as an optimal vaccination target due to the richness of antigen-presenting cells (i.e., langerhans cells, dermal dendritic cells, and dermal macrophages) in the dermal and epidermal layers, which are known to be involved in vaccine-induced immune responses (3). Additionally, the dense network of blood capillaries and lymphatic vessels within the dermis significantly facilitate the trafficking of leukocytes and dendritic cells, from the skin to the secondary lymphoid organs, thereby promoting the generation of a systemic humoral immune response (4, 5).

Needle-free injection (NFI) is an injection method that may eliminate the use of traditional capillary needles. It does not involve the penetration of flesh by a needle. Instead, it utilizes the instantaneous high pressure generated by a power source to propel the liquid vaccine through a nozzle, forming a high-speed, high-pressure jet (6). This process allows the vaccine to penetrate the outer layer of the skin, facilitating its delivery into the skin and releasing the therapeutic effects (6, 7). Needle-based injection (NI) vaccines typically create a liquid sphere within the dermis, whereas Needle-free injection disperse the vaccine more extensively throughout the skin tissue. The Needle-free injection increases contact between vaccine antigens and immune cells and has the potential to enhance the immune responses and reduce the vaccine dosage. Compared to needle injection, needle-free injection produces immune responses that are equivalent or stronger, capable of inducing higher antibody titers and serum antibody conversion rates. This advantage becomes particularly crucial during vaccine shortages, in the areas with limited vaccine resource, or an emergency vaccination satiation (8, 9).

In addition, the traditional Needle-based injection can lead to needle phobia, causing people to deviate from immunization schedules (10). Needle-based injection pose a risk of post-vaccination infections and iatrogenic transmission (11). Needle-free injection may overcome needle-associated fears, enhances vaccine compliance, eliminates the need for needle or syringe changes, reducing the risk of cross-contamination. Therefore the injection method simplifies medical waste disposal, thereby avoiding or minimizing the consequences of unsafe injections (12). Recently, needle-free immunization technology has been gradually promoted, and its application in the vaccine fields such as influenza vaccine, hepatitis B and other vaccines. The technology has become increasingly widespread due to its advantages over needle-based injections (13, 14). Mao et al. (15) studied the NFI of SARS-CoV-2-Spike-specific mRNA-Lipid Nanoparticle (LNP) vaccines and found that NFI mRNA-LNP vaccination is an effective alternative to traditional NI mRNA-LNP vaccination.

The recombinant novel coronavirus vaccine was built utilizing the adenovirus type-5 vector platform (16). It represents a non-replication adenovirus type-5 vector-based COVID-19 vaccine (Ad5-nCoV) expressing the spike protein of SARS-CoV-2. This vaccine, the first domestically approved adenovirus vector COVID-19 vaccine, has been included in the World Health Organization’s Emergency Use Listing (EUL) (17).

It induces both cellular and humoral immune responses against COVID-19 in the human body. Our early research has indicated that intramuscular injection of the Ad5-nCoV showed a higher incidence of local and systemic adverse reactions compared to inactivated vaccines (18). Additionally, studies have indicated that needle-free administration can reduce the occurrence of adverse events (19). However, there is currently no research on the use of needle-free injectors for Ad5-nCoV. Therefore, this study aims to explore the safety and immunogenicity of using a Needle-free injection of the Ad5-nCoV in animal experiments.

## 2 Materials and methods

### 2.1 Animal experiments

This study consisted of two different experiments, Study A and Study B. Study A consisted of 105 rats and the primary endpoint was to evaluate the safety and immunogenicity of two routes of vaccination with Ad5-nCoV. Study B included 32 rats as a supplementary experiment to observe only the pathological changes at the injection site after immunization. Laboratory investigations utilized female Sprague–Dawley (SD) rats meeting specific pathogen-free standards, with characteristics of 6–8 weeks of age and body mass ranging 200 ± 10 g, sourced through Jiangsu Huachuang Xinnuo Pharmaceutical Technology Co, Ltd. (Jiangsu, China). The specimens underwent housing adaptation within specialized chambers featuring precise environmental regulation (23 ± 1°C; 50 ± 10% relative humidity; photoperiod regime: 07:00–19:00 h illumination followed by darkness). Following a seven-day physiological adjustment interval, the experimental subjects were sustained with continuous availability of deionized food and water. The research methodology adhered to institutional protocols (validation code: 20221101030) as sanctioned by the Laboratory Animal Care and Use Committee of Southeast University, Nanjing, China.

### 2.2 Experimental vaccine

This novel immunological preparation emerged through collaborative efforts between the Beijing Institute of Biotechnology (Beijing, China) and CanSino Biologics (Tianjin, China). The immunogenic construct utilizes a replication-defective Ad5 delivery platform engineered to synthesize the spike glycoprotein of SARS-CoV-2. Each single-dose container houses a liquid formulation with a predetermined concentration of 5 × 10^10^ viral particles suspended in 0.5 mL volume. For administration purposes, a needle-free immunization system (POK-V-MBX) was acquired from Jiangsu Boke Technology Co., Ltd. (Taizhou, China). The needle-free injector utilizes a high-pressure jet generated by a dynamic power source, which propels the medicinal solution through a nozzle to create a high-velocity, high-pressure stream (typically exceeding 100 m/s). This enables the liquid to penetrate the epidermal barrier and infiltrate the intradermal tissue, with the entire process completed within 100 μs. The injection head’s frontal contact surface diameter is 0.14 mm, with a dose precision adjustable to 0.01 mL. The injector’s power source pressure can be modulated, facilitating precise vaccine administration into the intradermal, subcutaneous, and muscular tissues of rats models.

### 2.3 Vaccination of rats

The initial phase of investigation incorporated 105 SD rats undergoing a seven-day acclimation interval, after which systematic distribution yielded seven dose groups, each comprising 15 rats: the low-dose, medium-dose, and high-dose groups by needle-based intramuscular injection, and low-dose, medium-dose, and high-dose groups by needle-free intradermal injection; and a needle-free intradermal injection control group with saline. The quantitative parameters established a standardized delivery volume of 0.08 mL per test subject, this metric being derived through interspecies conversion algorithms incorporating surface area coefficients between human and rat subjects, while accounting for intradermal administration constraints in the rodent model (20, 21). The experimental dosing regimen implemented a four-fold incremental progression, with volumetric parameters set at: low-dose group (0.02 mL), medium-dose group (0.08 mL), and high-dose group (0.32 mL). The reference cohort underwent administration of 0.08 mL saline solution as vehicle control (Table 1). Study A was used for safety (body weight, Skin observation and histopathological examination) and immunogenicity. At day 28 after vaccination, the rats in study A were euthanized for SARS-CoV-2 pseudovirus neutralization assay, receptor binding domain (RBD) of SARS-CoV-2 spike glycoprotein and splenic T-cell response detection.

**Table 1:** Grouping table of rats.

Group	Vaccine	Injection dose (ml)	Immunization route	Injection site	Number
Study A					
NFI Low-dose group	Ad5-nCoV	0.02	Intradermal	Unilateral hindlimb	15
NFI Medium-dose group	Ad5-nCoV	0.08	Intradermal	Unilateral hindlimb	15
NFI High-dose group	Ad5-nCoV	0.32	Intradermal	Bilateral hind limbs and both sides of the back of the neck	15
NI Low-dose group	Ad5-nCoV	0.02	Intramuscular	Unilateral hindlimb	15
NI Medium-dose group	Ad5-nCoV	0.08	Intramuscular	Unilateral hindlimb	15
NI High-dose group	Ad5-nCoV	0.32	Intramuscular	Bilateral hindlimbs	15
NFI control group	Saline solution	0.08	Intradermal	Unilateral hindlimb	15
Study B					
NFI Low-dose group	Ad5-nCoV	0.02	Intradermal	Unilateral hindlimb	8
NFI Medium-dose group	Ad5-nCoV	0.08	Intradermal	Unilateral hindlimb	8
NI Low-dose group	Ad5-nCoV	0.02	Intramuscular	Unilateral hindlimb	8
NI Medium-dose group	Ad5-nCoV	0.08	Intramuscular	Unilateral hindlimb	8

NI, needle-based injection; NFI, needle-free injection.

In Study B, 32 SD rats were adaptively fed for 1 week and randomly assigned to four dose groups (*n* = 8 per group). SD rats were immunized needle-based intramuscularly or needle-free intradermally with 0.02 mL (low-dose) and 0.08 mL (middle-dose) of Ad5-nCoV. The rats in Study B were euthanized for the pathologic changes at the injection sites in the skin and muscle detection. To comply with ethical requirements for animal experimentation, euthanasia was performed by placing the animal in a euthanasia box and perfusing carbon dioxide at a rate of 10–30% chamber volume replacement per minute for approximately 5 min. The animal’s activity status was carefully monitored to ensure complete cessation of vital functions.

### 2.4 Skin observation and histopathological examination

In study A, the safety of 105 rats was evaluated on the day 28 after vaccination, focusing on body weight, local skin irritation and daily activities. The appearance, behavioral activity, injection site, mortality, and any other abnormalities of the rats were observed at least once within 2 h of inoculation, and then twice a day from day 2 until the end of the experiment. Skin irritation at the injection site was assessed within the first 7 days after immunization, and the occurrence of erythema and edema was recorded. The Draize dermal irritation scoring system (DDISS) was utilized to assess the irritation conditions, with scoring criteria outlined in Supplementary Table S1. Body weight was recorded daily for the first 7 days after vaccination, and then measured twice a week until the end of the study.

In Study A, the single-site injection doses were equal in the high-dose and medium-dose groups; therefore, Study B focused only on the pathologic changes at the injection sites in the skin and muscle in the low-dose and mid-dose groups. Euthanasia was performed at 1 h (1 h), 24 h (24 h), 48 h (48 h) and day 7 (7 d) after immunization, and two rats were euthanized at each time point. And 2–3 cm^2^ areas of the injection site’s epidermis and muscle tissue were excised and immersed in 4% paraformaldehyde for fixation. After routine paraffin embedding and sectioning, histopathological changes were observed under a microscope following hematoxylin and eosin staining.

### 2.5 SARS-CoV-2 pseudovirus neutralization assay

Serial blood specimens (0.1–0.2 mL per rat) were procured through jugular vein access at designated intervals (day 0, 7, 14, 21, and 28) post-immunization. Following serum isolation, neutralization capacity was evaluated against pseudovirus preparations. The analytical protocol assessed neutralizing antibodies (NAbs) targeting wild-type SARS-CoV-2, Delta and Omicron BA.4/5 subvariants via pseudovirus neutralization methodology, employing a threshold detection criterion of ≥1:30. Serum positivity was defined as a titer value ≥30, with values <30 considered negative. For negative samples (<30), the data were calculated using half of the cutoff value, which is 15. Neutralizing antibody titers were quantified using ND50 (the serum dilution required to neutralize 50% of viral infectivity). Seroconversion criteria stipulated a minimum four-fold elevation in neutralizing titers relative to baseline measurements.

After diluting rat serum, it was dispensed into a 96-well plate, with cell control and virus control groups established. Each sample was sequentially subjected to a threefold gradient dilution in 150 μL serum. The experimental procedure commenced with heat-inactivated sera undergoing sequential dilution, which was subsequently combined with pseudovirus preparations standardized to 2 × 10^4^ TCID50/ml. This mixture underwent thorough homogenization followed by thermal equilibration at 37°C for a duration of 60 min. The resulting preparation was introduced to ACE2-293 T cells maintained at 2 × 10^4^ cells/50 μL. The experimental setup utilized 96-well microplates, with duplicate wells receiving 50 μL of the cellular suspension. Terminal incubation proceeded in a 37°C CO_2_ environment for a 48-h duration. Following incubation, 100 μL of the supernatant was aspirated from each well in the 96-well plate, and 100 μL of Bio-Lite reporter gene assay reagent (Vazyme Medical Technology, Nanjing, China) was added. After a 3-min incubation period, chemiluminescence values (RLU) were measured using a microplate reader.

### 2.6 Enzyme linked immunosorbent (ELISA) assay

Quantitative assessment of wild-type SARS-CoV-2 RBD-specific IgG responses (RU/mL) was performed utilizing a commercial Anti-SARS-CoV-2 RBD-IgG ELISA kit (Vazyme Medical Technology, Nanjing, China), with analytical parameters establishing a cutoff titer of 1:800. The analytical workflow entailed the application of 100 μL serum onto antigen-coated microtitre surfaces, followed by a 60-min equilibration period and subsequent washing steps. A secondary incubation phase with antigen proceeded at 37°C for 30 min, succeeded by additional washing cycles. Spectrophotometric analysis at 450 nm was conducted following sequential introduction of substrate and termination reagents. Serum positivity was defined as an OD value ≥800, with values <800 considered negative. For negative samples (<800), the data were calculated using half of the cutoff value, which is 400.

### 2.7 Enzyme linked immunospot (ELISpot)

For cellular immunity evaluation, splenic tissue was harvested from 8 rats per group at day 28 post-immunization and maintained at −80°C. T-cell responses, specifically Interferon-*γ* (IFN-γ) production, were quantified through ex vivo Enzyme-linked Immuno-spot (ELISpot) methodology. SARS-CoV-2-specific cellular immune responses underwent assessment utilizing the commercial Rat IFN-γ ELISpot kit (Mabtech, Nacka Strand, Sweden) in accordance with vendor specifications.

The experimental protocol involved splenocyte preparations at 4 × 10^6^ cells/ml undergoing antigenic challenge with a peptide pool encompassing the full length spike glycoprotein. Cell cultures were maintained in pre-coated ELISpot plates under standardized conditions (37°C, 5% CO_2_, humidified atmosphere) for 20–24 h to evaluate T helper type 1 cytokine profiles (IFN-*γ*). The subsequent processing sequence comprised: PBS + 0.5% FBS rinses, 2-h exposure to Affinity-labeled antibodies, intermediate washing, 1-h ALP incubation, followed by BCIP/NBT chromogenic development. Color stabilization was achieved through extensive deionized water rinses, followed by light-protected desiccation. Spot enumeration employed an AID ELISPOT reader Classic, with results normalized to SARS-CoV-2-specific spots per 1 × 10^6^ splenocytes. Response positivity criteria stipulated minimum thresholds of five spot-forming cells per 1 × 10^5^ peripheral blood mononuclear cells coupled with a two-fold minimum elevation from baseline.

### 2.8 Statistical analysis

Statistical computations encompassed geometric mean titer (GMT) and geometric mean titer fold increase (GMFI) for both RBD-binding and neutralizing antibodies, incorporating two-sided 95% confidence intervals (CI) derived from t-distribution analysis of log-transformed titers. Categorical variable assessment employed *χ*^2^ methodology or Fisher’s exact test as dictated by data characteristics. Log-transformed antibody data underwent analysis of variance, while Wilcoxon rank-sum methodology was implemented for non-normal distributions. Seroconversion threshold criteria established a minimum four-fold elevation in antibody titers relative to baseline values. Data processing and visualization were executed using SPSS (version 25.0) and GraphPad Prism (version 8.0.2), with statistical significance threshold set at *p* < 0.05.

## 3 Results

### 3.1 Safety of NFI and NI

**Figure 1:**
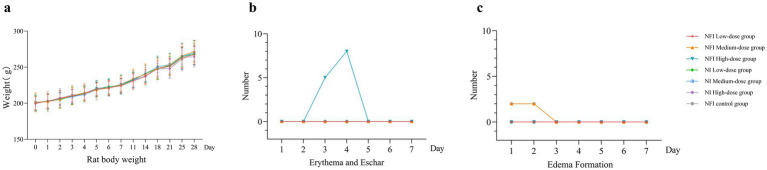
Trend of body weight change, erythema and eschar formation and edema formation in rats. Error bars are 95% CIs. Panel **(a)** is trend of body weight change 28 days after vaccination. Panel **(b)** shows changes in the number of erythema and eschar formation 7 days after vaccination. Panel **(c)** shows changes in the number of edema formation 7 days after vaccination.

The weights of each rat group were measured during the entire experimental duration, as illustrated in Figure 1. The weights of rats in all groups showed an increasing trend over the 28 days post-immunization. There was no significant difference in each vaccine group observed compared to the control group (Supplementary Table S2). These results indicate that immunization with varying doses of Ad5-nCoV via two administration routes does not impact the stable weight gain of rats.

The predominant skin reaction observed in rats post-needle-free vaccination was erythema. In the high-dose needle-free intradermal group, five rats exhibited localized erythema at day 3, eight rats at day 4 post-immunization, and erythema resolved at day 5 in these rates (Supplementary Table S3). No localized erythema was observed in the other groups. In the medium-dose needle-free intradermal group, two rats exhibited edema, which resolved at day 3 (Supplementary Table S3). In the NI control group, two rats experienced transient localized edema, and also resolved at day 3. No edema or erythema was observed locally in the intramuscular injection group. The intensity of all irritations was rated as “Score 1, very slight,” indicating that NI did not induce significant adverse effects on the inoculated local area.

**Figure 2:**
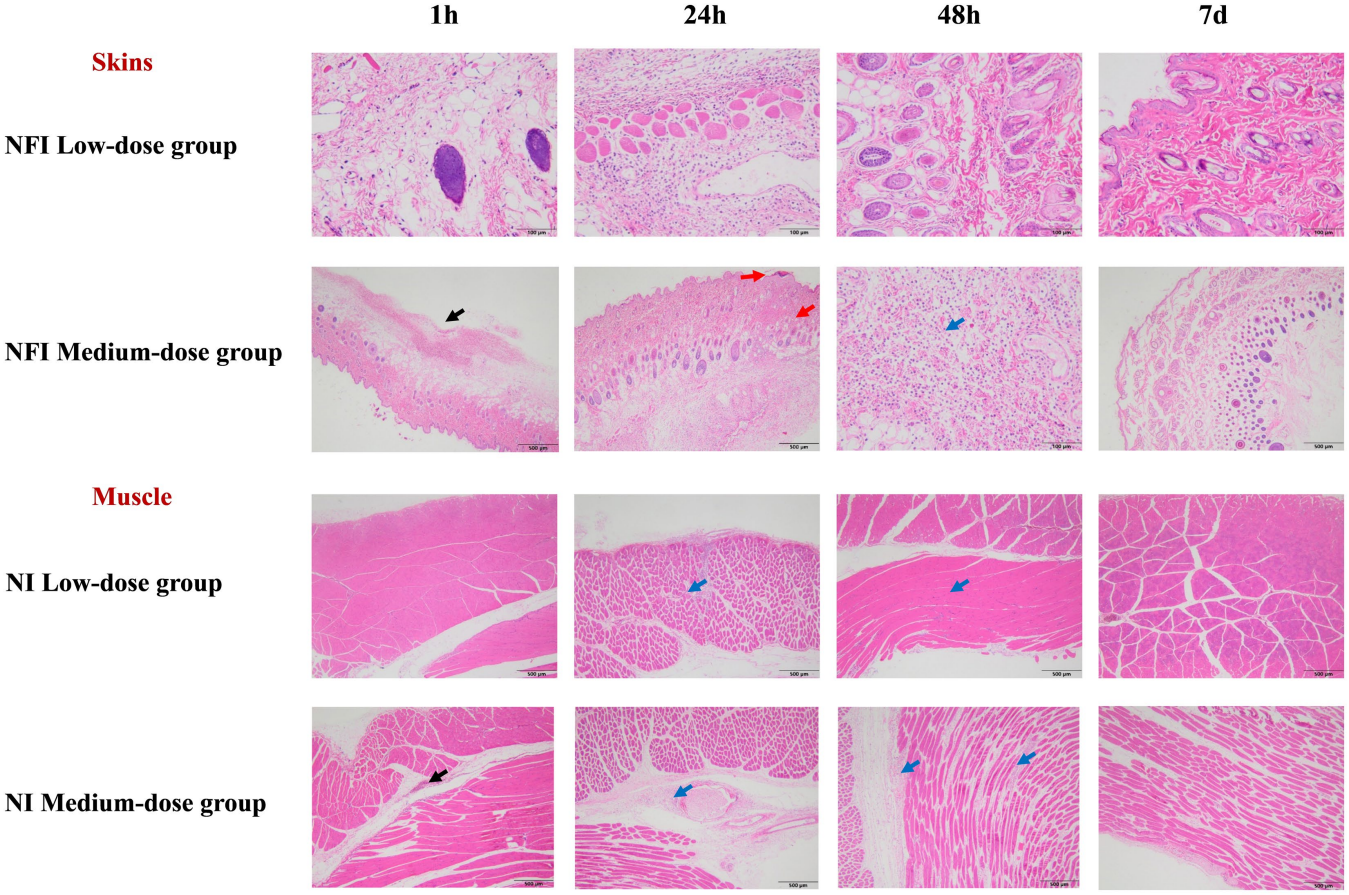
Skin and muscle histopathological examination. *The black arrows indicate mild to moderate local subcutaneous hemorrhage in the interstitium. The red arrows denote the formation of crusts in the epidermal cornified layer, with localized connective tissue proliferation visible in the dermis. The blue arrows signify mild inflammatory cell infiltration in the interstitium. Due to the equivalence in the dosage of the single-point injection between the high-dose group and the medium-dose group, the pathological changes in the skin and muscle were solely analyzed in the low and medium-dose groups.

Pathological findings of the skin and muscle at 1 h, 24 h, 48 h, and 7 d post-immunization were illustrated in Figure 2. No abnormal lesions were observed at any time point following low-dose intradermal injection. In the intramuscular injection of the medium dose, both with a needle and needle-free showed slight interstitial changes at 1 h and 24 h post-immunization, which slightly ameliorated by 48 h. At day 7 post-immunization, tissue alterations and local inflammatory reactions had fully resolved.

### 3.2 Neutralizing antibodies against wild-type SARS-CoV-2

**Figure 3:**
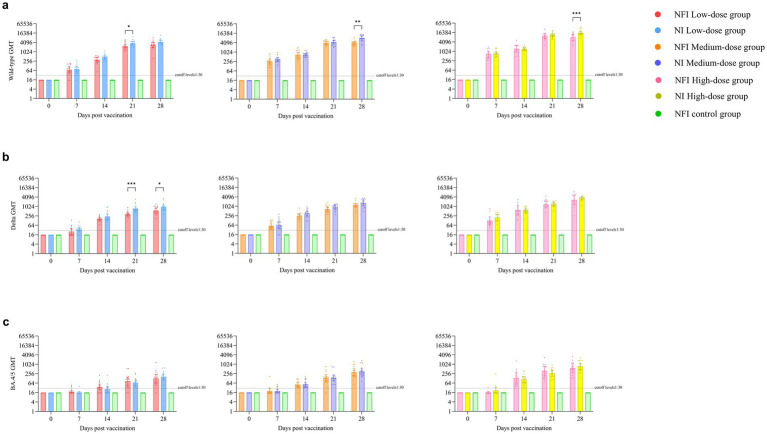
Pseudovirus neutralizing antibody levels against the wild-type strain, Delta subvariant and Omicron BA.4/5 subvariant. Error bars are 95% CIs. The horizontal dotted lines denote the cutoff levels for detection. *p < 0.05, **p < 0.01, ***p < 0.001. Panel **(a)** is pseudovirus neutralizing antibody levels against the wild-type strain. Panel **(b)** is pseudovirus neutralizing antibody levels against the Delta subvariant. Panel **(c)** is pseudovirus neutralizing antibody levels against the Omicron BA.4/5 subvariant.

The GMTs of neutralizing antibodies were below the detection limit for all groups at day 0 (Figure 3a). On day 7 post-immunization, the serum conversion rates were 60.00 and 66.67% for the low-dose NFI and NI groups, respectively, while the serum neutralizing antibody positive rates for medium- and high-dose groups reached 100% (Supplementary Table S4). By day 14 post-immunization, except for one case of non-seroconversion in the low-dose NFI group, all other groups of rats achieved seroconversion with a conversion rate of 100%, and the GMTs of neutralizing antibodies increased significantly. However, there were no difference between the low-dose, middle-dose or high-dose NI and NFI groups (*p* > 0.05) at day 14 post-immunization. At the day 14 post-immunization, the GMT in the low-dose NFI group increased to 302.10 (95% CI 215.88, 422.75), while in the low-dose NI group, it increased to 446.66 (348.04, 573.23). There was no statistical difference observed in neutralizing antibody levels between the two groups (*p* = 0.0548). Within the medium-dose group, the GMT for the NFI group was 684.11 (95% CI 469.10, 997.67), and for the NI group, it was 694.20 (95% CI 566.41, 850.82). Again, there was no statistical discrepancy in neutralizing antibody levels between these two groups (*p* = 0.9423). In the high-dose group, the GMT for the NFI group was 1478.34 (95% CI 923.13, 2367.49), and for the NI group, it was 1419.08 (95% CI 1180.63, 1705.68). Once more, there was no statistical variance observed in neutralizing antibody levels between these two groups (*p* = 0.8616). At day 28 post-immunization, GMT peaked in the all groups. In the medium-dose group, the NI group had significantly higher GMTs than the NFI group, with GMTs of 7970.57 (95% CI 5865.04, 10831.96) and 4007.66 (95% CI 3235.22, 4964.53), respectively, showing a statistically significant difference between the two groups (*p* = 0.0004). Similarly, in the high-dose group, the NI group had significantly higher GMTs than the NFI group (*p* = 0.0047), with GMTs of 15403.34 (95% CI 12005.74, 19762.46) and 7945.8 (95% CI 5239.29, 12050.65), respectively. There were no statistically significant differences between the needle-based and NFI methods in the low-dose group (*p* = 0.0590).

### 3.3 Neutralizing antibodies against Delta subvariant

Before vaccination, the serum antibody levels against the Delta strain were below the detection limit in all groups (Supplementary Table S5). At day 14 post-immunization, all rat sera exhibited seroconversion, with a seroconversion rate of 100%. No statistically significant differences were observed between the groups on days 7 and 14 post-immunization (Figure 3b). At day 28, neutralizing antibody levels against the Delta strain peaked in all groups. In the low-dose regimen, the NI group showed a higher GMT than the NFI group (*p* = 0.0356), with GMTs of 906.10 (95%CI 645.34, 1272.22) and 557.10 (95%CI 401.01, 773.96), respectively. Compared to GMTs against the original strain, the neutralizing antibody levels against the Delta strain at day 28 were 4.8–5.1 times lower in the low-dose group, 3.4–4.8 times lower in the medium-dose group, and 3.0–4.7 times lower in the high-dose group (Supplementary Figure S1).

### 3.4 Neutralizing antibodies against Omicron BA.4/5 subvariant

The neutralizing antibody results against the Omicron BA.4/5 revealed that at day 7 post-immunization, except for 1 rat each in the NFI medium-dose group and NI high-dose group showing seroconversion, no seroconversion occurred in the other groups (Supplementary Table S6). At day 0 and day 7 post-immunization, the GMTs of neutralizing antibodies in all groups were below the cutoff levels. At days 14 and 21, there was an increase in the GMTs of neutralizing antibodies compared to NFI control group in all groups (Figure 3c). At day 28, the titers of neutralizing antibodies peaked in all groups. In the low-dose group, the neutralizing antibody levels against the BA.4/5 strains were 22.6–26.7 times lower than those against the wild-type strain and 4.4–5.6 times lower than those against the Delta strain. In the medium-dose group, the levels were 12.8–22.7 times lower than those against the wild-type strain and 3.7–4.7 times lower than those against the Delta strain. In the high-dose group, the levels were 13.5–20.5 times lower than those against the wild-type strain and 4.4–4.6 times lower than those against the Delta strain (Supplementary Figure S1).

### 3.5 Wild-type SARS-CoV-2 RBD-specific IgG antibodies

At day 0, the levels of S-RBD IgG antibodies in all groups were below the cutoff levels (Supplementary Figure S2). At day14, except for one case of non-seroconversion in the NFI low-dose group, all other groups exhibited a seroconversion rate of 100%. At day 14, in the low-dose group, the NFI control group exhibited higher S-RBD IgG antibody levels compared to the NI group (*p* = 0.0176). No statistically significant differences were observed between the two immunization methods in the low and medium dose groups. At day 28, the S-RBD IgG antibody levels peaked in all dose groups. In the high-dose group, the NI group exhibited significantly higher S-RBD IgG antibody levels compared to the NFI control group (*p* = 0.0220). Trend analyses revealed statistically significant differences in antibody levels over time across all groups (*p* < 0.0001).

### 3.6 The IFN-γ-secreting cells per million cells on day 28 post-immunization

**Figure 4:**
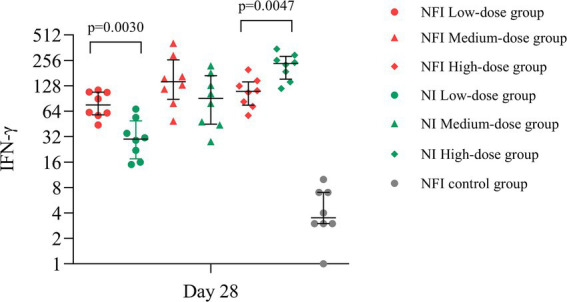
The IFN-γ-secreting cells per million cells on day 28 post-immunization. Error bars indicate median and quartiles of IFN-γ-secreting cells per million cells for each group; scatter points indicate IFN-γ-secreting cells per million cells for each sample.

Based on the ELISpot assay, at day 28, significant vaccine-induced specific T-cell responses were observed across all experimental groups. The median number of IFN-γ-secreting cells per million cells in all groups were markedly higher than those in the control group (*p* < 0.0001) (Figure 4). In the low-dose group, the NFI control group exhibited higher median number of IFN-γ-secreting cells compared to the NI group (*p* = 0.0030), with median values of 76.00 (IQR 58.00, 107.75) and 30.00 (IQR 17.50, 49.25) IFN-*γ*-secreting cells per million cells, respectively, (Supplementary Table S7). Within the medium-dose group, the median number of IFN-γ-secreting cells were 143.00 (IQR 88.50, 260.00) for the NFI group and 91.00 (IQR 45.00, 168.00) for the NI group, with no statistically significant difference observed between the two group (*p* = 0.1691). In the high-dose group, the NI group demonstrated a higher median number of IFN-γ-secreting cells compared to the NFI group (*p* = 0.0047), with median values of 234.50 (IQR 153.05, 285.50) and 109.50 (IQR 75.50, 142.00) IFN-γ-secreting cells per million cells, respectively.

## 4 Discussion

Our results supported the needle-free intradermal immunization would be considered as an alternative immunization method to replace the traditional needle-based intramuscular injection for the Ad5-nCoV. The results indicate that both needle-free intradermal injection and needle-based intramuscular injection of the adenovirus type 5 vectored COVID-19 vaccine demonstrate good safety profiles in rats. Immunogenicity studies reveal that, at equivalent doses, needle-free intradermal injection and needle-based intramuscular injection of the Ad5 vaccine induce comparable immune responses.

Following immunization with Ad5-nCoV, rats exhibited normal activity and weight gain, Histopathological analysis with no significant differences observed among two immunization routes. Intradermal Needle-free injection exhibited transient irritancy to rat skin, resolving within 7 days. The findings indicate that post-vaccine immunization does not induce persistent cutaneous inflammation or tissue damage at the rat injection site, demonstrating concordance with the animal experimental observations reported by Zhu et al. (22). Multiple clinical trials have reported that local adverse reactions were more common with intradermal injection than intramuscular injection, while systemic adverse reactions were less frequently reported, the underlying mechanism may be attributed to the intradermal injection’s high density of immunocompetent cells and rich vascular network within the skin tissue, which enables a more direct activation of the local immune system, consequently resulting in more pronounced local adverse reactions (1, 2, 23). Further validation in the human population is required to assess the adverse reactions associated with needle-free vaccination.

The level of neutralizing antibodies serves as a crucial indicator for assessing vaccine immunogenicity. In pseudovirus neutralization assays targeting the wild-type SARS-CoV-2, immune responses were induced by both Needle-free injection and Needle-based injection at day 14 post-immunization, with no significant difference observed between the two methods. Tawinprai et al. (2) demonstrated that at day 14 post-immunization, individuals who received two doses of CoronaVac showed similar neutralizing antibody levels with a third dose of ChAdOx1 nCoV-19 vaccine administered intradermally at 40% of the standard dose compared to a third dose administered intramuscularly at the standard dose. During vaccine shortages, in the areas with limited vaccine resource, or an emergency vaccination satiation, the intradermal vaccination strategy may contribute to conserving vaccine supplies.

Pseudovirus neutralization assays targeting the Delta variant revealed that both vaccination methods significantly elicited immune responses at day 14 post-immunization. In comparison to the wild-type strain, neutralizing antibody levels against the Delta variant were slightly diminished, aligning with trends observed in population studies (24). Our research indicated that both Needle-based injection and Needle-free injection in various dosage groups elicited immune responses against the Omicron BA.4/5 subvariant. At day 28, the serum antibody seroconversion rate exceeded 90%, and the neutralizing antibody levels against BA.4/5 in the intradermal Needle-free injection group were comparable to those in the intramuscular Needle-based injection group.

The specificity of T-cell responses is crucial for directly targeting and eliminating virus-infected cells (25). Detection of IFN-*γ*-secreting cells per million cells has become a key method for assessing cellular immune efficacy, with its secretion serving as a critical indicator of cellular immune. Through ELISpot experiments, we observed that, at day 28, the Ad5-nCoV could significantly induce the number of IFN-γ-secreting cells compared to the control group. This suggested that both immunization methods can stimulate the generation of specific T-cell responses. Our results indicated differences in the number of IFN-γ-secreting cells produced between the two immunization methods across various dosage groups. In the low-dose group, intradermal Needle-free injection induced higher number of IFN-γ-secreting cells compared to intramuscular Needle-based injection. This aligned with Tawinprai et al.’s (2) study. However, in the high-dose regimen of our study, intramuscular injection resulted in higher number of IFN-γ-secreting cells than intradermal Needle-free injection. Sophonmanee et al.’s (26) study also observed higher number of IFN-γ-secreting cells after vaccine administration in the muscle compared to intradermal injection.

This study has certain limitations that warrant cautious interpretation of the results. Firstly, the safety observations and the immunogenicity assessment in the experiment only extended for 28 days, lacking coverage over a more extended time frame. Additionally, safety and immunogenicity experiments for the needle-free intradermal injection vaccine were exclusively conducted in rats, necessitating further investigation in primates or human populations in the future. The protective efficacy of the vaccine against SARS-CoV-2 was not directly evaluated through viral challenge experiments in animal models, which warrants further investigation in future studies.

In conclusion, our results in rats demonstrate that both intradermal Needle-free injection and intramuscular Needle-based injection of the Ad5-nCoV exhibited favorable safety profiles, and comparable robust immunogenicity at equivalent dosages. Our findings supported needle-free immunization would as a viable alternative to needle-based injection. Further in-depth studies are warranted for a comprehensive exploration of the long-term immunogenicity associated with needle-free intradermal injection.

## Data Availability

The raw data supporting the conclusions of this article will be made available by the authors, without undue reservation.
