# Supplementary material for: Safety and immunogenicity of Ad5-nCoV administered intradermally by needle-free injector in rats
Source: Front Med (Lausanne). 2025 Feb 14;12:1543398. doi: 10.3389/fmed.2025.1543398 (PMC11867966; doi:10.3389/fmed.2025.1543398)
Supplement: Supplementary file 1 [file Supplementary_file_1.docx]

## ***Supplementary Material***

## **Table S1. Draize dermal irritation scoring system (DDISS).**

| Score | Erythema and Eschar Formation | Edema Formation |
| --- | --- | --- |
| 0 | No erythema | No edema |
| 1 | Very slight erythema (barely  perceptible) | Very slight erythema (barely perceptible) |
| 2 | Well-defined erythema | Slight edema (edges of area well defined bydefinite raising) |
| 3 | Moderate to severe erythema Value | Moderate edema(raised approximately 1 mm) |
| 4 | Severe erythema (beet redness)to slight eschar formation(injuries in depth) | Severe edema (raised >1 mmand extending beyond area of exposure) |

## **Table S2. Rat body weight recording table (Mean±SD, g, n=15).**

| Day | NFI  Low-dose group | NFI Medium-dose group | NFI  High-dose group | NI  Low-dose group | NI Medium-dose group | NI  High-dose group | NFI  control group | p value |
| --- | --- | --- | --- | --- | --- | --- | --- | --- |
| Day 0 | 199.84±8.95 | 201.14±13.04 | 200.22±10.78 | 200.02±9.78 | 200.49±11.07 | 199.43±10.26 | 201.79±12.34 | 0.9979 |
| Day 1 | 202.77±9.83 | 202.05±11.43 | 202.13±10.59 | 203.01±9.98 | 202.39±9.82 | 203.09±9.09 | 202.47±13.81 | 1.0000 |
| Day 2 | 207.16±10.79 | 205.25±12.50 | 206.33±8.82 | 204.77±10.76 | 205.29±8.02 | 206.66±8.15 | 206.93±13.27 | 0.9937 |
| Day 3 | 209.89±9.37 | 210.31±12.49 | 209.23±9.76 | 209.86±11.43 | 209.00±9.88 | 210.57±8.59 | 211.54±12.68 | 0.9967 |
| Day 4 | 212.93±8.43 | 214.48±12.43 | 212.82±10.90 | 213.43±9.31 | 211.69±9.91 | 214.13±10.43 | 213.23±11.57 | 0.9946 |
| Day 5 | 219.89±8.94 | 218.21±10.36 | 220.71±10.68 | 219.54±9.81 | 218.64±9.39 | 220.07±11.03 | 218.07±13.13 | 0.9911 |
| Day 6 | 221.35±9.25 | 221.17±10.18 | 221.77±11.37 | 223.27±10.33 | 220.89±9.62 | 221.53±10.53 | 221.87±12.00 | 0.9979 |
| Day 7 | 224.16±10.25 | 226.07±12.07 | 226.18±13.02 | 224.17±9.63 | 225.76±8.99 | 224.73±9.07 | 225.02±12.84 | 0.9970 |
| Day 11 | 230.75±9.81 | 233.91±13.19 | 232.79±10.80 | 232.41±10.20 | 232.04±9.68 | 231.65±10.58 | 231.85±13.46 | 0.9941 |
| Day 14 | 237.22±9.47 | 240.03±14.32 | 240.71±11.99 | 241.13±11.58 | 237.46±7.05 | 237.63±12.15 | 240.20±12.57 | 0.9234 |
| Day 18 | 248.09±10.61 | 247.81±15.06 | 250.41±14.85 | 248.33±14.74 | 248.64±6.17 | 248.67±14.37 | 247.35±14.37 | 0.9979 |
| Day 21 | 251.03±10.92 | 253.33±14.82 | 253.55±15.47 | 251.61±13.22 | 252.27±10.55 | 247.82±12.82 | 248.02±13.54 | 0.8263 |
| Day 25 | 262.85±14.92 | 265.63±16.47 | 264.93±17.98 | 264.10±12.96 | 265.24±11.15 | 263.19±15.69 | 260.73±15.85 | 0.9833 |
| Day 28 | 267.14±13.14 | 271.75±14.74 | 270.27±16.81 | 268.17±14.26 | 268.44±10.50 | 264.45±14.02 | 269.37±16.81 | 0.8815 |

NI=Needle-based injection. NFI=needle-free injection.

## **Table S3. Erythema and edema formation record sheet.**

| Group | Day 1 | Day 2 | Day 3 | Day 4 | Day 5 | Day 6 | Day 7 |
| --- | --- | --- | --- | --- | --- | --- | --- |
| Erythema and Eschar |  |  |  |  |  |  |  |
| NFI Low-dose group | 0 | 0 | 0 | 0 | 0 | 0 | 0 |
| NFI Medium-dose group | 0 | 0 | 0 | 0 | 0 | 0 | 0 |
| NFI High-dose group | 0 | 0 | 5 | 8 | 0 | 0 | 0 |
| NI Low-dose group | 0 | 0 | 0 | 0 | 0 | 0 | 0 |
| NI Medium-dose group | 0 | 0 | 0 | 0 | 0 | 0 | 0 |
| NI High-dose group | 0 | 0 | 0 | 0 | 0 | 0 | 0 |
| NFI control group | 0 | 0 | 0 | 0 | 0 | 0 | 0 |
| Edema Formation |  |  |  |  |  |  |  |
| NFI Low-dose group | 0 | 0 | 0 | 0 | 0 | 0 | 0 |
| NFI Medium-dose group | 2 | 2 | 0 | 0 | 0 | 0 | 0 |
| NFI High-dose group | 0 | 0 | 0 | 0 | 0 | 0 | 0 |
| NI Low-dose group | 0 | 0 | 0 | 0 | 0 | 0 | 0 |
| NI Medium-dose group | 0 | 0 | 0 | 0 | 0 | 0 | 0 |
| NI High-dose group | 0 | 0 | 0 | 0 | 0 | 0 | 0 |
| NFI control group | 2 | 2 | 0 | 0 | 0 | 0 | 0 |

Data are number of erythema and edema cases in each group. NI=Needle-based injection. NFI=needle-free injection.

## **Table S4. GMT, seroconversion rate, and GMFI of neutralizing antibodies to** **wild-type SARS-CoV-2 pseudovirus.**

|  | NFI Low-dose group (N=15) | NI Low-dose group (N=15) | p value | NFI Medium-dose group(N=15) | NI Medium-dose group(N=15) | p value | NFI High-dose group(N=12) | NI High-dose group(N=15) | p value | NFI control group(N=15) |
| --- | --- | --- | --- | --- | --- | --- | --- | --- | --- | --- |
| Day 0 |  |  |  |  |  |  |  |  |  |  |
| GMT | 15.00(-) | 15.00(-) | - | 15.00(-) | 15.00(-) | - | 15.00(-) | 15.00(-) | - | 15.00(-) |
| Day 7 |  |  |  |  |  |  |  |  |  |  |
| GMT | 69.56(44.48, 108.78) | 77.01(52.05, 113.93) | 0.7162 | 253.58(180.17, 356.90) | 332.66(252.45, 438.35) | 0.1957 | 664.71(430.66, 1026.00) | 662.35(441.52, 993.62) | 0.7918 | 15.00(-) |
| Seroconversion | 60.00(32.28, 83.66) | 66.67(38.38, 88.18) | >0.9999 | 100(-) | 100(-) | - | 100(-) | 100(-) | - | 0 |
| GMFI | 4.64(2.97, 7.25) | 5.13(3.47, 7.60) | 0.7816 | 16.91(12.01, 23.79) | 22.18(16.83, 29.22) | 0.1873 | 44.31(28.71, 68.40) | 44.16(29.44, 66.24) | 0.7918 | 1.00(-) |
| Day 14 |  |  |  |  |  |  |  |  |  |  |
| GMT | 302.10(215.88, 422.75) | 446.66(348.04, 573.23) | 0.0548 | 684.11(469.10, 997.67) | 694.20(566.41, 850.82) | 0.9423 | 1478.34(923.13, 2367.49) | 1419.08(1180.63, 1705.68) | 0.8616 | 15.00(-) |
| Seroconversion | 93.33(68.05, 99.83) | 100(-) | >0.9999 | 100(-) | 100(-) | - | 100(-) | 100(-) | - | 0 |
| GMFI | 20.14(14.39, 28.18) | 29.78(23.20, 38.22) | 0.0548 | 45.61(31.27, 66.51) | 46.28(37.76, 56.72) | 0.9423 | 98.56(61.54, 157.83) | 94.61(78.71, 113.71) | 0.8616 | 1.00(-) |
| Day 21 |  |  |  |  |  |  |  |  |  |  |
| GMT | 2342.37(1742.79, 3148.22) | 3544.88(2740.42, 4585.50) | 0.0313 | 3949.18(3286.28, 4745.78) | 4404.36(3372.08, 5752.65) | 0.4764 | 9208.52(6308.23, 13442.24) | 11054.11(8825.64, 13845.26) | 0.3531 | 15.00(-) |
| Seroconversion | 100(-) | 100(-) | - | 100(-) | 100(-) | - | 100(-) | 100(-) | - | 0 |
| GMFI | 156.16(116.19, 209.88) | 236.33(182.69, 305.70) | 0.0313 | 263.28(219.09, 316.39) | 293.62(224.81, 383.51) | 0.4764 | 613.90(420.55, 896.15) | 736.94(588.38, 923.02) | 0.3531 | 1.00(-) |
| Day 28 |  |  |  |  |  |  |  |  |  |  |
| GMT | 2848.76(1935.31, 4193.36) | 4312.68(3413.63, 5448.51) | 0.0590 | 4007.66(3235.22, 4964.53) | 7970.57(5865.04, 10831.96) | 0.0004 | 7945.87(5239.29, 12050.65) | 15403.34(12005.74, 19762.46) | 0.0047 | 15.00(-) |
| Seroconversion | 100(-) | 100(-) | - | 100(-) | 100(-) | - | 100(-) | 100(-) | - | 0 |
| GMFI | 189.92(129.02, 279.56) | 287.51(227.58, 363.23) | 0.0590 | 267.18(215.68, 330.97) | 531.37(391.00, 722.13) | 0.0004 | 529.73(349.29, 803.38) | 1026.89(800.38, 1317.50) | 0.0047 | 1.00(-) |
| F value | 111.67 | 129.10 |  | 213.05 | 183.63 |  | 75.08 | 171.87 |  |  |
| p value* | <0.0001 | <0.0001 |  | <0.0001 | <0.0001 |  | <0.0001 | <0.0001 |  |  |

N = number of rats. Data are GMT (95% CI), seroconversion (%, 95%CI) or GMFI (95% CI). GMT=geometric mean titer. GMFI=geometric mean fold increase. NI=Needle-based injection. NFI=needle-free injection. Seroconversion was defined as proportion of participants with at least a four-fold increase of Wild-type pseudovirus compared to the baseline level before the boost. *The p-value indicates the results of trend analysis of variance comparing each time point.

## **Table S5. GMT, seroconversion rate, and GMFI of neutralizing antibodies to Delta subvariant pseudovirus.**

|  | NFI Low-dose group (N=15) | NI Low-dose group (N=15) | p value | NFI Medium-dose group(N=15) | NI Medium-dose group(N=15) | p value | NFI High-dose group(N=12) | NI High-dose group(N=15) | p value | NFI control group(N=15) |
| --- | --- | --- | --- | --- | --- | --- | --- | --- | --- | --- |
| Day 0 |  |  |  |  |  |  |  |  |  |  |
| GMT | 15.00(-) | 15.00(-) | - | 15.00(-) | 15.00(-) | - | 15.00(-) | 15.00(-) | - | 15.00(-) |
| Day 7 |  |  |  |  |  |  |  |  |  |  |
| GMT | 24.99(16.82, 37.13) | 33.29(24.21, 45.79) | 0.2245 | 57.19(42.37, 77.21) | 66.12(40.48, 107.99) | 0.5190 | 126.64(73.96, 216.86) | 199.47(130.80, 304.17) | 0.0951 | 15.00(-) |
| Seroconversion | 13.33(1.66, 40.46) | 6.67(0.17, 31.95) | >0.9999 | 33.33(11.82, 61.62) | 46.67(21.27, 73.41) | 0.7104 | 91.67(61.52, 99.79) | 93.33(68.05, 99.83) | >0.9999 | 0 |
| GMFI | 1.67(1.12, 2.48) | 2.22(1.61, 3.05) | 0.2245 | 3.81(2.82, 5.15) | 4.41(2.70, 7.20) | 0.5190 | 8.44(4.93, 14.46) | 13.30(8.72, 20.28) | 0.0951 | 1.00(-) |
| Day 14 |  |  |  |  |  |  |  |  |  |  |
| GMT | 161.06(131.86, 196.73) | 233.54(156.51, 348.48) | 0.4547 | 248.52(187.73, 329.00) | 352.65(251.03, 495.42) | 0.0996 | 599.89(323.84, 1111.28) | 556.56(420.75, 736.21) | 0.8114 | 15.00(-) |
| Seroconversion | 100(-) | 100(-) | - | 100(-) | 100(-) | - | 100(-) | 100(-) | - | 0 |
| GMFI | 10.74(8.79, 13.12) | 15.57(10.43, 23.23) | 0.0548 | 16.57(12.52, 21.93) | 23.51(16.74, 33.03) | 0.9423 | 39.99(21.59, 74.09) | 37.10(28.05, 49.08) | 0.8616 | 1.00(-) |
| Day 21 |  |  |  |  |  |  |  |  |  |  |
| GMT | 314.77(241.28, 410.64) | 712.36(506.12, 1002.64) | 0.0003 | 663.90(488.15, 902.92) | 879.38(681.22, 1135.19) | 0.1427 | 1395.25(840.28, 2316.76) | 1353.67(1054.91, 1737.05) | 0.9081 | 15.00(-) |
| Seroconversion | 100(-) | 100(-) | - | 100(-) | 100(-) | - | 100(-) | 100(-) | - | 0 |
| GMFI | 20.98(16.09, 27.38) | 47.49(33.74, 66.84) | 0.0003 | 44.26(32.54, 60.20) | 58.63(45.41, 75.68) | 0.1427 | 93.02(56.02, 154.45) | 90.25(70.33, 115.80) | 0.9081 | 1.00(-) |
| Day 28 |  |  |  |  |  |  |  |  |  |  |
| GMT | 557.10(401.01, 773.96) | 906.10(645.34, 1272.22) | 0.0356 | 1174.25(886.69, 1555.06) | 1658.85(1170.80, 2350.36) | 0.1058 | 2684.12(1393.59, 5169.76) | 3297.34(2679.05, 4058.34) | 0.5223 | 15.00(-) |
| Seroconversion | 100(-) | 100(-) | - | 100(-) | 100(-) | - | 100(-) | 100(-) | - | 0 |
| GMFI | 37.14(26.73, 51.60) | 60.41(43.02, 84.82) | 0.0356 | 78.28(59.11, 103.67) | 110.59(78.05, 156.69) | 0.1058 | 178.94(92.91, 344.65) | 219.82(178.60, 270.56) | 0.5223 | 1.00(-) |
| F value | 110.93 | 53.64 |  | 116.38 | 140.54 |  | 51.03 | 369.67 |  |  |
| p value* | <0.0001 | <0.0001 |  | <0.0001 | <0.0001 |  | <0.0001 | <0.0001 |  |  |

N = number of rats. Data are GMT (95% CI), seroconversion (%, 95%CI) or GMFI (95% CI). GMT=geometric mean titer. GMFI=geometric mean fold increase. NI=Needle-based injection. NFI=needle-free injection. Seroconversion was defined as proportion of participants with at least a four-fold increase of Delta antibodies subvariant pseudovirus compared to the baseline level before the boost. *The p-value indicates the results of trend analysis of variance comparing each time point.

## **Table S6. GMT, seroconversion rate, and GMFI of neutralizing antibodies to Omicron BA.4/5** **subvariant pseudovirus.**

|  | NFI Low-dose group (N=15) | NI Low-dose group (N=15) | p value | NFI Medium-dose group(N=15) | NI Medium-dose group(N=15) | p value | NFI High-dose group(N=12) | NI High-dose group(N=15) | p value | NFI control group(N=15) |
| --- | --- | --- | --- | --- | --- | --- | --- | --- | --- | --- |
| Day 0 |  |  |  |  |  |  |  |  |  |  |
| GMT | 15.00(-) | 15.00(-) | - | 15.00(-) | 15.00(-) | - | 15.00(-) | 15.00(-) | - | 15.00(-) |
| Day 7 |  |  |  |  |  |  |  |  |  |  |
| GMT | 18.09(14.51, 22.56) | 15.99(13.95, 18.33) | 0.4732 | 19.47(13.60, 27.88) | 19.34(15.08, 24.81) | 0.7211 | 16.10(13.78, 18.80) | 21.49(14.11, 32.71) | 0.2201 | 15.00(-) |
| Seroconversion | 0 | 0 | - | 6.67(0.17, 31.95) | 0 | >0.9999 | 0 | 6.67(0.17, 31.95) | >0.9999 | 0 |
| GMFI | 1.21(0.97, 1.50) | 1.07(0.93, 1.22) | 0.4732 | 1.30(0.91, 1.86) | 1.29(1.01, 1.65) | 0.7211 | 1.07(0.92, 1.25) | 1.43(0.94, 2.18) | 0.2201 | 1.00(-) |
| Day 14 |  |  |  |  |  |  |  |  |  |  |
| GMT | 35.47(22.79, 55.20) | 26.13(17.22, 39.66) | 0.2416 | 50.18(36.51, 68.95) | 53.06(38.92, 72.34) | 0.8787 | 134.17(62.71, 287.07) | 110.44(73.14, 166.77) | 0.8392 | 15.00(-) |
| Seroconversion | 26.67(7.79, 55.10) | 13.33(1.66, 40.46) | 0.6513 | 33.33(11.82, 61.62) | 33.33(11.82, 61.62) |  | 58.33(27.67, 84.83) | 80.00(51.91, 95.67) | 0.3981 | 0 |
| GMFI | 2.36(1.52, 3.68) | 1.74(1.15, 2.64) | 0.2416 | 3.35(2.43, 4.60) | 3.54(2.59, 4.82) | 0.8787 | 8.94(4.18, 19.14) | 7.36(4.88, 11.12) | 0.6089 | 1.00(-) |
| Day 21 |  |  |  |  |  |  |  |  |  |  |
| GMT | 84.66(46.28, 154.85) | 67.65(43.89, 104.29) | 0.5226 | 134.23(81.57, 220.89) | 136.19(87.89, 211.04) | 0.9630 | 381.76(187.81, 776.00) | 274.39(180.82, 416.36) | 0.3683 | 15.00(-) |
| Seroconversion | 60.00(32.28, 83.66) | 53.33(26.59, 78.73) | >0.9999 | 93.33(68.05, 99.83) | 80.00(51.91, 95.67) | 0.5977 | 100(-) | 100(-) | - | 0 |
| GMFI | 5.64(3.09, 10.32) | 4.51(2.93, 6.95) | 0.5226 | 8.95(5.44, 14.73) | 9.08(5.86, 14.07) | 0.9630 | 25.45(12.52, 51.73) | 18.29(12.06, 27.76) | 0.3683 | 1.00(-) |
| Day 28 |  |  |  |  |  |  |  |  |  |  |
| GMT | 126.18(69.29, 229.75) | 161.36(104.76, 248.54) | 0.4811 | 313.32(190.99, 514.02) | 351.19(214.01, 576.31) | 0.7289 | 586.76(308.65, 1115.44) | 750.55(465.29, 1210.71) | 0.5011 | 15.00(-) |
| Seroconversion | 80.00(51.91, 95.67) | 86.67(59.54， 98.34) | >0.9999 | 93.33(68.05, 99.83) | 100(-) | >0.9999 | 100(-) | 100(-) | - | 0 |
| GMFI | 8.41(4.62, 15.32) | 10.76(6.98, 16.57) | 0.4811 | 20.89(12.73, 34.27) | 23.41(14.27, 38.42) | 0.7289 | 39.12(20.58, 74.36) | 50.04(31.02, 80.71) | 0.5011 | 1.00(-) |
| F value | 16.94 | 43.08 |  | 39.65 | 34.05 |  | 22.91 | 64.06 |  |  |
| p value* | 0.0001 | <0.0001 |  | <0.0001 | <0.0001 |  | <0.0001 | <0.0001 |  |  |

N = number of rats. Data are GMT (95% CI), seroconversion (%, 95%CI) or GMFI (95% CI). GMT=geometric mean titer. GMFI=geometric mean fold increase. NI=Needle-based injection. NFI=needle-free injection. Seroconversion was defined as proportion of participants with at least a four-fold increase of omicron BA.4/5 antibodies subvariant pseudovirus compared to the baseline level before the boost. *The p-value indicates the results of trend analysis of variance comparing each time point.

## **Table S7.The IFN-γ-secreting cells per million cells** **on day 28 post-immunization**

|  | NFI Low-dose group (N=15) | NI Low-dose group (N=15) | p value | NFI Medium-dose group(N=15) | NI Medium-dose group(N=15) | p value | NFI High-dose group(N=12) | NI High-dose group(N=15) | p value | NFI control group(N=15) |
| --- | --- | --- | --- | --- | --- | --- | --- | --- | --- | --- |
| Median(IQR) | 76.00(58.00, 107.75) | 30.0(17.50, 49.25) | 0.0030 | 143.00(88.50, 260.00) | 91.00(45.00, 168.00) | 0.1691 | 109.50(75.50, 142.00) | 234.50(153.50, 285.50) | 0.0047 | 3.50(3.00, 7.00) |

N = number of rats. Data are median (Q1, Q3). NI=Needle-based injection. NFI=needle-free injection.

## **Figure S1. Comparison of neutralising antibody levels among strains 28 days after immunization**


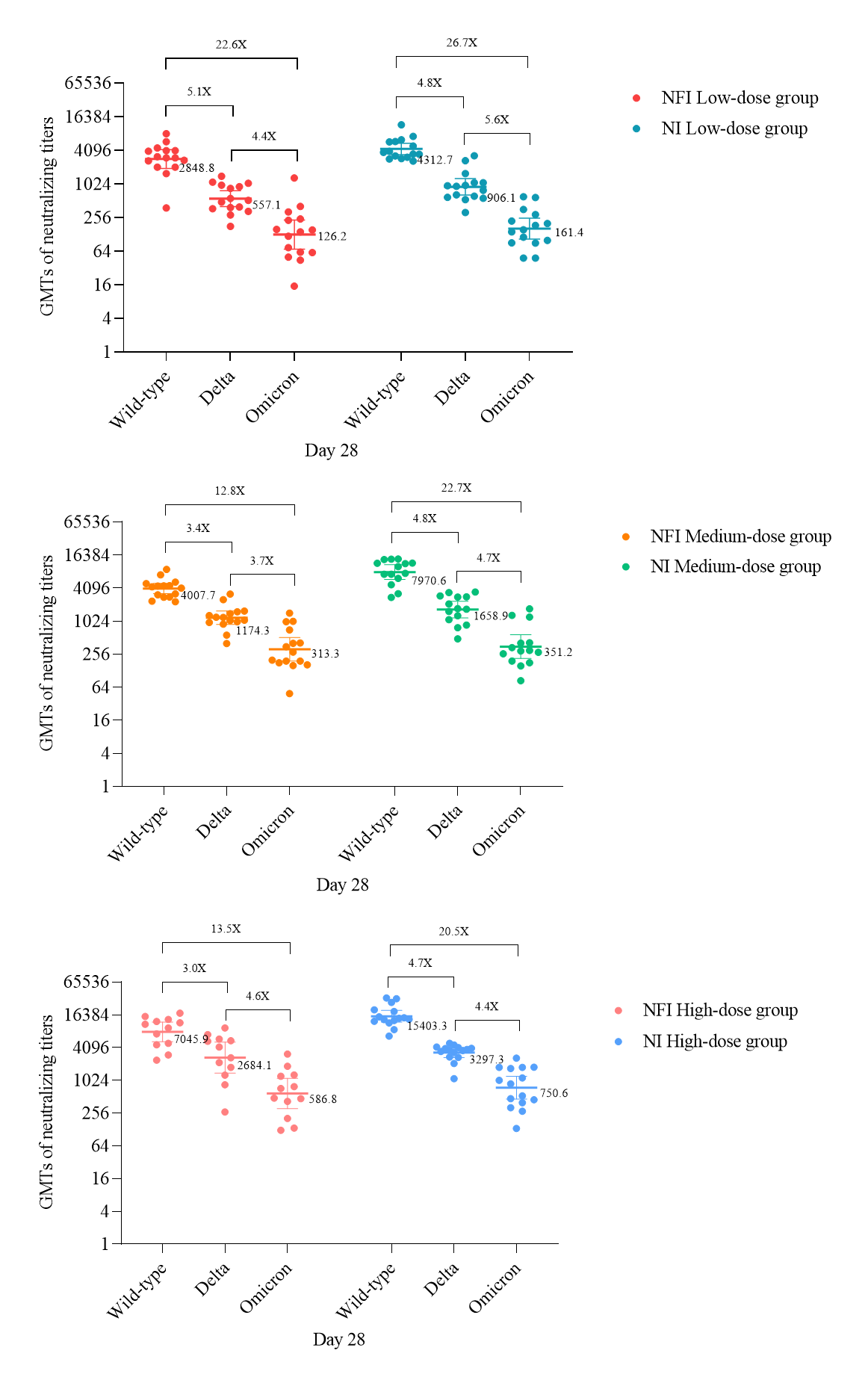


GMT=geometric mean titers; the number to the right of the scatter is the estimated GMT for each group; X indicates the multiplicative difference between the two groups.

## **Figure S2. Wild-type SARS-CoV-2 RBD-specific IgG antibodies**


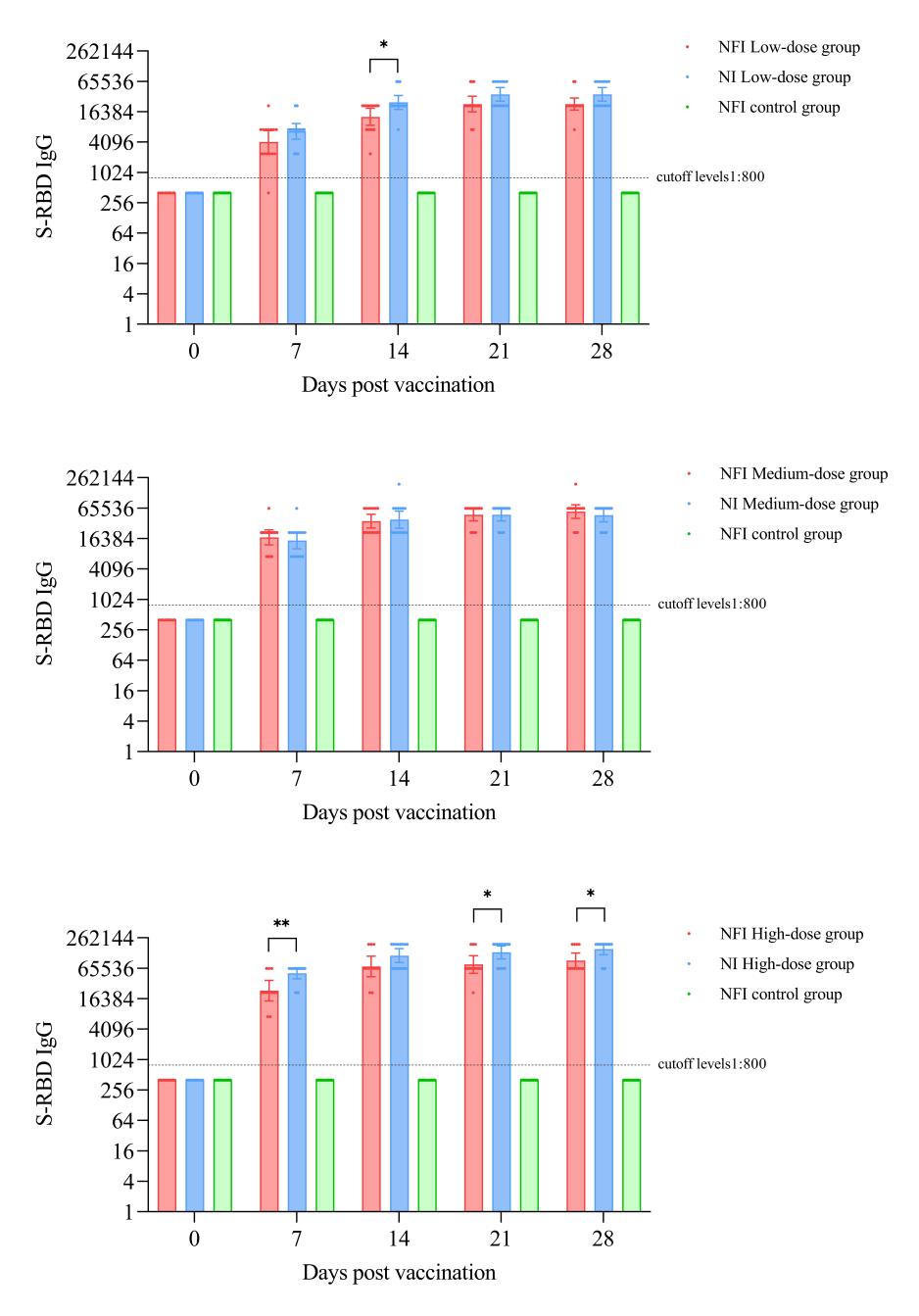


Error bars are 95% CIs. The horizontal dotted lines denote the cutoff levels for detection. RBD-IgG = receptor-binding domain (RBD)-specific IgG antibodies.* P<0.05，** P<0.01.
